# Determining the Dormancy Type of the Endangered *Linum mulleri* by Testing 7000 Seeds

**DOI:** 10.3390/plants14070984

**Published:** 2025-03-21

**Authors:** Ludovica Dessì, Marco Porceddu, Lina Podda, Alba Cuena Lombraña, Gianluigi Bacchetta

**Affiliations:** Sardinian Germplasm Bank (BG-SAR), Centre for Conservation of Biodiversity (CCB), Department of Life and Environmental Sciences, University of Cagliari, Viale Sant’Ignazio da Laconi 9-13, 09123 Cagliari, Italy; ludovica.dessi@unica.it (L.D.); lina.podda@unica.it (L.P.); alba.cuena@unica.it (A.C.L.); bacchet@unica.it (G.B.)

**Keywords:** *Linum*, endemic plant, seed germination, dormancy, pre-treatment

## Abstract

*Linum mulleri* is an endemic taxon of southwestern Sardinia (Italy), categorised as Endangered (EN) on the IUCN Red List and included in Annexes II and IV of the Habitats Directive (92/43/EEC) as priority species for conservation. This study investigated the germination ecophysiology of *L. mulleri* and the possible presence of dormancy by using 7000 seeds, providing useful information for conservation strategies. The germination response of fresh seeds was evaluated under different temperatures, photoperiods, pre-treatments [cold stratification (C); warm stratification (W); W+C; C+W+C; dry after-ripening (DAR)], and different gibberellic acid (GA_3_) concentrations. *L. mulleri* germinated under controlled conditions, particularly at 15 and 20 °C, while germination percentages (GP) never exceeded 5% at 5 and 30 °C. C and C+W+C induced secondary dormancy, delaying germination, whereas W, DAR, and GA_3_ stimulate it. Light and dark incubation showed no significant differences in regards to GP. W, DAR, and 250 mg/L GA_3_ effectively overcame physiological dormancy (PD), expanding the germination temperature range to below 10 and above 25 °C. These responses suggested type 3 non-deep PD, as germination temperatures extended from a moderate range to both low and high temperatures. Analyzing 7000 seeds provided crucial information regarding dormancy and germination strategies, supporting both ex situ and in situ conservation efforts.

## 1. Introduction

Seed germination is a delicate event which determines the establishment of a plant, contributing to the persistence of the population [1]. Seed germination requirements are species-specific, and they are also influenced by various factors such as temperature, humidity, and light [2]. In seasonal climates and wet soils, temperature is usually the main environmental factor influencing seed germination [3], and each species has a temperature range within which germination can occur [4]. Sometimes, germination may behave differently, depending on whether the temperature is constant or alternating [5,6]. When no germination occurs under a wide range of conditions over a period of more than four weeks, the seeds likely exhibit dormancy. Five main classes of seed dormancy are recognized [7], and, among them, physiological dormancy (PD) is the most common form worldwide [7]. PD seeds contain a physiological inhibiting mechanism in the embryo that prevents radicle emergence [7,8], distinguished into three levels, according to the conditions required to break it and promote germination: non-deep (divided into types one through six, depending on the temperature range for dormancy break), intermediate, and deep [9].

*Linum* L. is the most well-known genus among the 22 belonging to the Linaceae family; it is distributed in temperate regions, primarily in the Northern Hemisphere, although 14 species are known in the Cape Region of South Africa and a similar species is found in New Zealand [10]. It is estimated that there are about 230 species belonging to the *Linum* genus [11,12,13]. One of the distribution centres of the *Linum* genus is the Mediterranean area, with 75 species, along with India, where it likely originated in the northwestern region, then spreading to Ethiopia, the Fertile Crescent, and Russia [10,14]. In Italy, the *Linum* genus is represented by 27 taxa [15]; among them, 10 are native to Sardinia, and only *Linum mulleri* Moris is reported as endemic to the island [16]. The species belonging to this genus mainly grow on rocks or in well-drained limestone or sandy soils [10,14] and are divided into five subsections: *Linum* L., *Dasylinum* (Planch.) Juz., *Syllinum* Griseb., *Cathartolinum* Griseb., and *Linastrum* (Planch.) Bentham [17]. *Linum* species are annual or perennial herbs, generally erect with hard bark; the flowers can be blue, red, yellow, or white, borne on axillary or terminal racemes [14]. The genus has some economic relevance, as it is cultivated for the production of seed oil and fibres, particularly for the species *L. usitatissimum* L. (common flax) [18]. Some studies have been conducted on the germination ecophysiology of *Linum* seeds, primarily focused on *L. usitatissimum*, whose seeds do not exhibit dormancy [19,20,21], while the literature data on other *Linum* species appear to be scarce. It has generally been shown that *Linum* seeds can exhibit dormancy, and germination requires a period of cold stratification or post-maturation immediately after dispersal. For example, seeds of *L. perenne* L. were able to break dormancy after a period of cold stratification [22]; *L. radiola* L. seeds required an after-ripening period [23]; *L. olympicum* Boiss. was able to germinate under both light and dark conditions, with GA_3_ and/or cold stratification [24]; while *L. catharticum* L. seeds are light-dependent and require a period of cold treatment [25,26].

In this work, we focused our attention on studying the germination ecophysiology of *L. mulleri*, an exclusively endemic plant of southwestern Sardinia, particularly in the Iglesiente biogeographic subsector. This species exhibits an unfavourable conservation status due to the fragility of the habitat in which it grows, its small size, and the isolation of its populations. Additionally, the environmental restoration activities of abandoned mining sites pose a further risk to some areas within the range of *L. mulleri* [27]. The high risk of extinction has led to *L. mulleri* being listed as Endangered (EN) on the IUCN Red List [28]. The taxon has been included in Annex II and IV of the Habitats Directive (92/43/EEC) as a priority species for conservation. This taxon is included among the species studied in the LIFE SEEDFORCE—LIFE20 NAT/IT/001468 (Using SEED banks to restore and reinFORCE the endangered native plants of Italy) project, which aims to improve the conservation status of 29 plants species listed in Annex II of the Habitats Directive (92/43/EEC).

Plant conservation requires a thorough understanding of the plant life cycle; for these reasons, determining the germination characteristics of the seeds of these species under study is essential to contribute to this purpose. Currently, to our knowledge, there is no information available in the literature regarding the ecophysiology of the germination of *L. mulleri*. Based on existing literature for the genus *Linum* [23,24,25,26], we hypothesized that the seeds of *L*. *mulleri* might also exhibit dormancy. To explore this hypothesis, the objectives of this study were to evaluate the germination response of seeds to (I) different temperatures and photoperiods, (II) several pre-treatments (warm and/or cold stratification, dry after-ripening), and (III) under various concentrations of gibberellic acid (GA_3_). To achieve these objectives, controlled laboratory experiments were conducted; understanding the germination behaviour and determining whether dormancy is present and if so, determining its class and type, are useful for implementing more effective management and conservation measures for this species.

## 2. Results

### 2.1. Seed Germination

**Figure 1:**
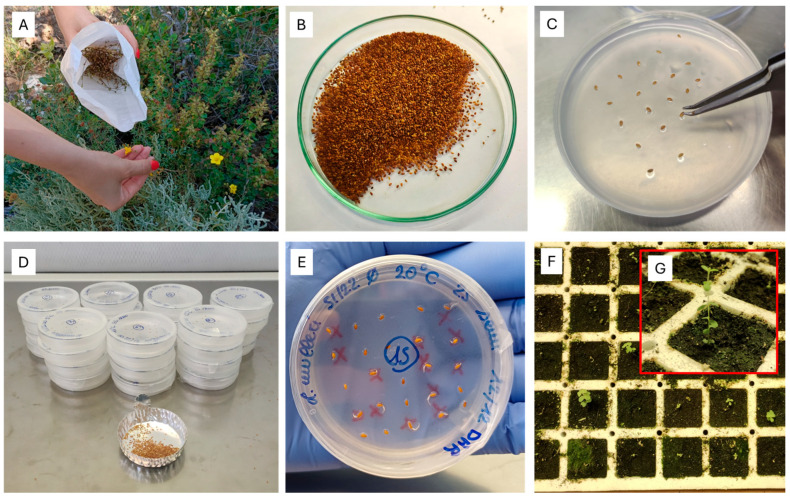
(**A**) Germplasm collection of *Linum mulleri*; (**B**) seed lot after cleaning, with removal of impurities and empty seeds; (**C**) sowing seeds in 1% agar substrate; (**D**) Petri dishes sealed with Parafilm and ready for incubation in growth chambers; (**E**) example of germinated seeds at 20 °C after DAR; (**F**) seedlings of *Linum mulleri* in the soil substrate; (**G**) detail of *Linum mulleri* seedling.

In general, the percentage of contaminated *L. mulleri* seeds during the germination test was less than 1–2%, and the percentage of non-viable seeds at the end of the experiments was generally less than 10%. Germinated seeds (see example in Figure 1E of germinated seeds at 20 °C after DAR) with radicles approximately 0.2 to 0.5 mm long were removed from Petri dishes to provide plant material for subsequent in situ translocation (Figure 1F,G). Petri dishes were replaced when necessary (i.e., at the end of each pretreatment cycle and at the start of all treatments). Furthermore, no evident detrimental contamination of the seeds or agar that could have compromised seed viability during the germination test was observed.

### 2.2. Germination During Pre-Treatment

**Figure 2:**
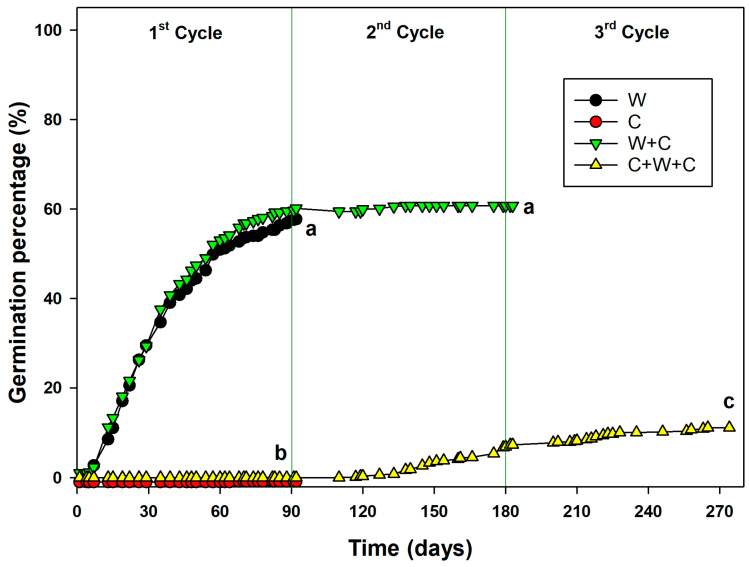
Germination percentage during pre-treatments. I, II, and III correspond to the three pretreatment cycles applied to *Linum mulleri* seeds, with I = 90 days for W (warm stratification) and C (cold stratification), II = 180 days for W+C (warm stratification followed by cold stratification), and III = 270 days for C+W+C (cold stratification followed by warm stratification and another cold stratification period). Data represent the mean of four replicates (±SD). The GLMs were applied during the germination, and values with the same letter are not statistically different by post hoc pairwise *t*-test comparisons.

During the pre-treatments of W, C, W+C, and C+W+C, germination occurred. According to the GLM results, the type of pre-treatment applied showed statistically significant differences (*p* < 0.05) in regards to seed germination. Seeds exposed to W and W+C recorded germination percentage that reached up to 59% (W up to 58.72% and W+C up to 59.14%). In both pre-treatments, germination occurred during the first 90 days; in the case of W+C, during the W cycle, the germination reached 60%, while after 90 days (i.e., during the C cycle), the increase was very limited, bringing the final germination (after 180 days) to 60.75% (Figure 2). Seeds under C recorded a very low germination percentage, even below 1% (0.14%), whereas seeds subjected to C+W+C recorded germination percentages of 0% for the first 90 days (first C cycle), 6.87% from the 90th to the 180th day (W cycle), and a final germination percentage of 11.14% after 270 days (second C cycle) (Figure 2).

### 2.3. Effect of Photoperiod, Treatment, and Pre-Treatment on Seed Germination

The incubation temperature (T), the treatment and pre-treatment (Tr), and the interaction of these two factors (T × Tr) had a significant effect on GP (*p* < 0.001) (Table 1).

The post hoc analysis indicated that there were no statistical differences in the GP response of seeds incubated under dark conditions compared to all other treatments and pre-treatments applied (Table 2). The W and DAR conditions were statistical different in GP compared to those for C+W+C, while the results for C and W+C were statistically different only when compared to those for DAR (Table 2). The incubation seeds under CTR conditions, however, reveal statistical differences compared to the results for C+W+C (Table 2).

**Figure 3:**
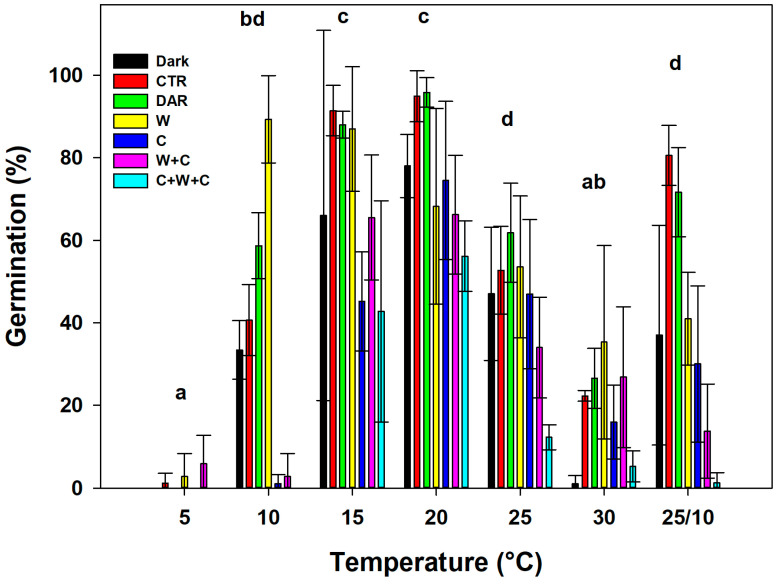
Germination percentage (GP) of *Linum mulleri* seeds under different treatments and pre-treatments [CTR (control), C (cold stratification), W (warm stratification), W+C (warm stratification followed by cold stratification), C+W+C (cold stratification followed by warm stratification and another cold stratification period), DAR (dry after-ripening), and dark] and incubated at constant (5, 10, 15, 20, 25, 30 °C) and alternating (25/10 °C) temperatures. Data represent the mean of four replicates (±SD). GLMs were carried out on the germination, and values with the same letter are not statistically different by post hoc pairwise *t*-test comparisons.

At a temperature of 5 °C, the highest GP (5%) was achieved in seeds that had previously undergone a W+C period; for all other treatments and pre-treatments, the GP was equal to or slightly above 0% (Figure 3). At 10 °C, the W recorded the highest GP (89.3%), while seeds previously incubated at C showed low germination capacity, not exceeding 5%, along with seeds that underwent W+C (around 5%). Seeds incubated under light and dark conditions achieved a GP of 40.62% and 33.37%, respectively. The DAR revealed a GP of 58.65% (Figure 3). At 15 and 20 °C, the highest germination percentages were recorded, with GP exceeding 40%, regardless of the treatment or pre-treatment considered. The lowest GP (42.23%) was recorded for seeds that had previously undergone C+W+C treatment at 15 °C. The highest GP (95.82%) was recorded for seeds that had previously undergone DAR and were then incubated at 20 °C. At 25 °C, the highest GP was recorded for seeds that had previously undergone DAR, with a percentage of 61.82%, while the lowest GP was recorded for seeds that had previously undergone C+W+C (12.25%) (Figure 3). At 30 °C, as at 5 °C, the GPs were consistently low, with percentages not exceeding 35%, regardless of the treatment and pre-treatment considered (Figure 3). The alternating temperature of 25/10 °C for seeds incubated under light recorded a GP of 80.57%, while, as also seen at other temperatures, seeds previously incubated with the C+W+C treatment had a lower GP compared to the results for all other treatments and pre-treatments (1.2%) (Figure 3).

### 2.4. Effect of Gibberellic Acid (GA_3_) on Seed Germination

The concentration (Co), the incubation temperature (T), and the interaction of these two factors (Co × T) showed significant effects on GP (*p* < 0.001) (Table 3).

**Figure 4:**
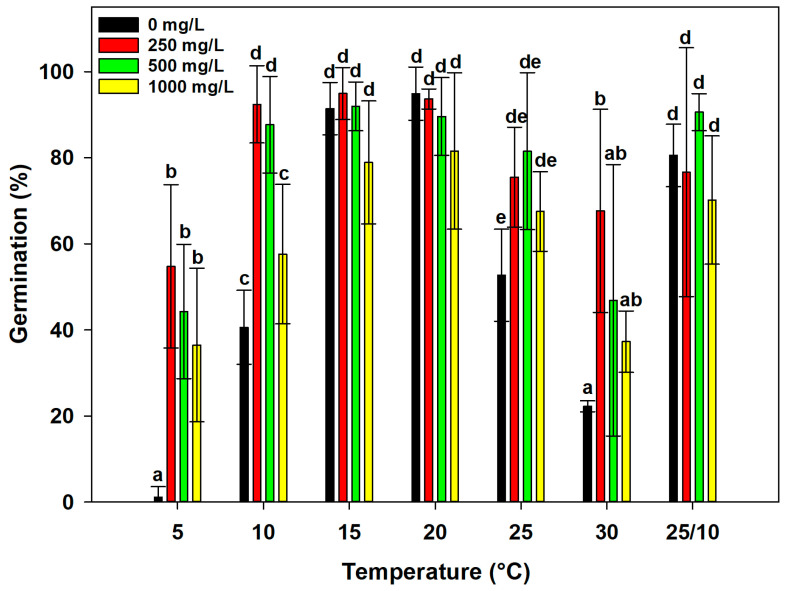
Effect of different concentrations of GA_3_ (0, 250, 500, 1000 mg/L) on seed germination of *Linum mulleri* incubated at constant (5, 10, 15, 20, 25, and 30 °C) and alternating (25/10 °C) temperatures. The data represent the mean of four replicates (±SD). Values with the same letter are not statistically different by post hoc pairwise *t*-test comparisons.

At temperatures of 15, 20, and 25/10 °C, the concentrations behave in the same way, recording germination percentages above 60% (Figure 4). At a temperature of 10 °C, the concentration of 0 mg/L of GA_3_ recorded the lowest germination percentage (40.6%), while other concentrations were always above 50%; in particular, the concentration of 250 mg/L of GA_3_ recorded GP of 92.39%, 500 mg/L of GA_3_ recorded GP of 87.65%, and 1000 mg/L of GA_3_ recorded GP of 57.59% (Figure 4). The temperatures of 5 and 30 °C resulted in a lower GP. In particular, the GP at 5 °C reached 54.4% at a concentration of 250 mg/L of GA_3_, while at a concentration of 0 mg/L of GA_3_, the GP did not exceed 2%. At 30 °C, again, the concentration of 0 mg/L of GA_3_ recorded the lowest GP (22.3%), while 250 mg/L of GA_3_ recorded the highest GP (66.67%) (Figure 4).

### 2.5. Rate and Widening of Germination Temperature

The seeds treated with CTR, W, and DAR reached T_50_ in less time compared to the other pre-treatments (C, W+C, and C+W+C) at all tested temperatures (Table 4). At temperatures of 15 and 20 °C, notably lower T_50_ values were recorded compared to the results for the other temperatures and pre-treatments tested. At 15 °C, W showed the fastest germination (T_50_ = 5 days) compared to the results for CTR and DAR (T_50_ = 9 ± 1 and 8 ± 1, respectively). At 20 °C, DAR showed the fastest germination, with a T_50_ of 8 ± 2 days, compared to 8 ± 4 for CTR and 14 ± 16 days for W. Even at 10 °C, W and DAR achieved 50% germination, a result not achieved with the other pre-treatments and treatments applied to the seeds. The values obtained were 7 ± 2 for W and 12 ± 1 days for DAR (Table 4).

Regarding the GA_3_ treatment, the 250 mg/L concentration recorded the lowest T_50_ values at all tested temperatures compared to the results for other concentrations. The only exception occurred at 10 °C, where the 500 mg/L concentration showed a T_50_ of 15 ± 1 compared to 16 ± 1 days for the 250 mg/L concentration (Table 4).

**Figure 5:**
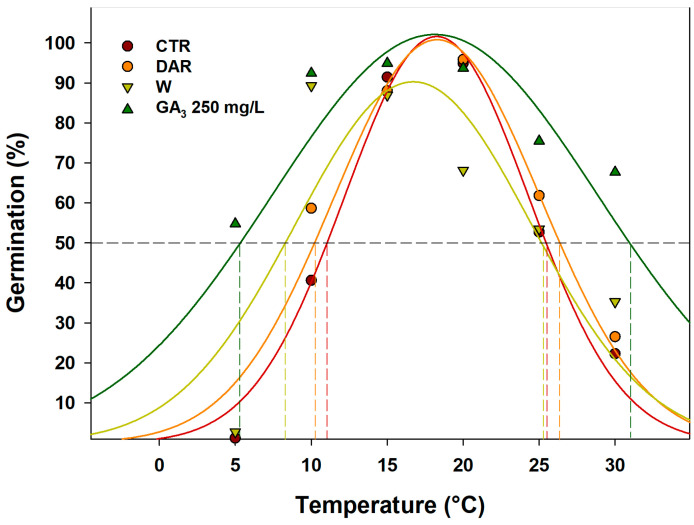
Gaussian curves representing the effects of CTR (control), W (warm stratification), DAR (dry after-ripening), and 250 mg/L of GA_3_ on the germination dynamics of *Linum mulleri* seeds. Points correspond to actual data, and solid lines indicate the fitted lines from Gaussian regressions. Vertical dotted lines indicate the minimum temperature at which a 50% germination rate can be achieved under the different treatments. The data represent the mean of four replicates.

Figure 5 shows the correlation among germination percentages and incubation temperatures, considering the treatments and pre-treatments for which germination rates equal to or greater than 50% were observed (CTR, DAR, W, and 250 mg/L of GA_3_), using a Gaussian curve. In these treatments, the results highlighted an extension of the temperatures at which 50% germination could be achieved. In CTR, temperatures extended from a minimum of approximately 12 °C up to a maximum of 26 °C. In the DAR pre-treatment, the range expands from about 11 °C to 27 °C, while in the W pre-treatment, the range extends from about 8 °C to 25.5 °C. Seeds sown with 250 mg/L of GA_3_ showed the greatest extension of temperature range, with a range from approximately a minimum of 5 °C to a maximum of 32 °C.

## 3. Discussion

This study demonstrates that *L. mulleri* was able to produce seeds capable of germinating under controlled conditions, in particular at 15 and 20 °C.

Despite the long incubation period, the percentage of contaminated *L. mulleri* seeds during the germination test was less than 1–2%, and seed mortality detected at the end of the germination tests remained below 10%; this aspect suggested that, processing the seeds following meticulous protocols, the absence of disinfection treatments did not negatively affect the germination outcomes in *L. mulleri* seeds. This result aligns with the findings of Ref. [2], who stated that fully developed and healthy seeds are generally not attacked by fungi and bacteria, whereas lower-quality seeds are more susceptible to such infections. Furthermore, the same authors highlight that fungal contamination is typically a problem for fruits rather than for smooth seeds, such as those of *L. mulleri*, further reducing the risk of infection during incubation. No previous germination studies on different *Linum* spp. have reported seed contamination [19,20,21,22,23,24,25,26], and in very few experiments, the seeds were preliminary surface-sterilized using sodium hypochlorite solution [21,24]. This behaviour could also lead to the hypothesis that *Linum* spp. seeds, including *L. mulleri*, may boast some type of seed defence system, i.e., some protective chemicals in their seed coats; thus, further specific study is required regarding this aspect.

The germination rates observed in *L. mulleri* during pre-treatments, as in the W, C, W+C, and C+W+C phases, is attributed principally to the effect of high incubation temperatures (i.e., 25 °C). In fact, the GP achieved during moist warm stratification (W) and warm followed by cold stratification (W+C), with over 50% in both pre-treatments, differed from the GP obtained during cold stratification (C) and with the pre-treatments starting with a cold stratification (C+W+C) (less than 1% in both pre-treatments). The incubation temperature of seeds is a factor that significantly influences the germination phase, either inhibiting or promoting the physiological processes involved in radicle emergence [29]. Incubation temperature also significantly influenced seed germination after the application of treatments and pre-treatments, under both light and dark conditions. The lowest germination percentage, but the highest percentage of ungerminated and viable seeds, was recorded at 5 °C and 30 °C. This limitation in seed germination at too high or too low temperatures could represent an ecological advantage, as germination is prevented under unfavourable climatic conditions, allowing germination to commence when temperatures are milder [2,30]. Low germination percentage and rates were also observed in seeds that underwent a cold stratification process before being incubated at other incubation temperatures. In many species, cold and moist conditions act as mechanisms to delay seed germination until the end of winter, when more favourable conditions arise [29,31]. C and C+W+C pre-treatments in seeds of *L. mulleri* seem to impose a secondary dormancy, delaying seed germination, even at low temperatures, after these cycles of pre-treatments with respect to W and DAR, which also stimulate germination at lower temperatures. This response could be a sign of the presence of physiological dormancy (PD) [2]. This behaviour provides an ecological advantage for seeds, allowing germination to be completed at milder temperatures [2]. The highest GP (more than 60%) was recorded at temperatures of 15 and 20 °C. In a Mediterranean climate, such as the one in which *L. mulleri* grows, this behaviour is quite common. This characteristic is identified as the “Mediterranean germination syndrome” hypothesis [32,33], which ensures complete germination during the early spring or autumn rainy seasons and protects young plants from exposure to summer drought (e.g., [32,33,34,35,36]). The ability to germinate even at alternating temperatures of 25/10 °C, in both treated and untreated seeds, might indicate that germination may also occur in the superficial soil layers, where the influence of alternating temperatures is greater [5,37]. This suggests that this taxon grows in climatic areas with significant differences in day and night temperatures. These results are consistent with several studies reporting a positive effect of fluctuating temperature regimes on seed germination percentage (e.g., [6,38]) and can partly explain the germination niche of a species and thus, its habitat requirements and distribution [6]. Contrary to what is reported in the literature for some *Linum* species [23,25,26], *L. mulleri* does not depend strictly on light for seed germination, as seed incubation under light or dark conditions showed no significant differences in GP. The ability of *L. mulleri* seeds to germinate under both light and dark conditions and at alternating temperatures highlights their great adaptability to different environmental conditions.

It is known that GA_3_ stimulates germination, especially in species that exhibit dormancy [39,40]. For example, in *L. olympicum* [24], GA_3_ at different concentrations (250, 500, and 1000 mg/L) was used as a substitute for cold stratification, proving particularly effective for breaking dormancy at a concentration of 1000 mg/L. In our study, GA_3_ stimulated seed germination by widening the temperature range. At the temperature of 5 °C, the 0 mg/L GA_3_ concentration recorded a percentage of less than 2%, while at 250 mg/L GA_3_, it reached as high as 54.4%. The same occurred at the temperature of 30 °C. These germination behaviors are in line with those noted previous studies that demonstrated the capacity of GA_3_ to enhance germination by overcoming PD [41,42].

In agreement with the work of Refs. [24,43], our study highlighted how different concentrations of GA_3_ have different effects on germination, emphasizing the importance of testing various concentrations. Higher concentrations of GA_3_ can inhibit germination [43], a result consistent with our observation that concentrations greater than 250 mg/L were less effective in promoting seed germination. These findings support the idea that optimal concentrations must be determined for each species to avoid negative effects and maximize germination success.

Based on the results obtained, it is possible to affirm that GA_3_ at a concentration of 250 mg/L was capable of breaking a form of PD in seeds of *L. mulleri*, expanding the range of temperatures at which this species is able to germinate, both for temperatures below 10 °C and for those above 25 °C. Freshly matured seeds with non-deep PD can either germinate over only a very narrow range of temperatures or cannot germinate at any temperature [2], and in the presence of non-deep PD, it is possible to break dormancy using warm or cold stratification or by using GA_3_ [7,44]. The use of GA_3_ was detected as an effective strategy to overcome PD and promote germination in other endangered species, especially in species exhibiting non-deep PD [41,45].

The W, DAR, and GA_3_ treatments in seeds of *L. mulleri* have also proven to be effective for breaking dormancy at lower and higher temperatures and consequently, for widening the temperature range of germination, especially when the temperature drops below 15 and 20 °C. Accordingly, the seeds of *L. mulleri* display a non-deep PD. In agreement with our results, *L. radiola* [23] requires an after-ripening period of 30 °C for 28 days to break dormancy; in contrast, in the literature, many authors have reported the positive effect of cold stratification on breaking seeds dormancy in other species belonging to the genus *Linum* (see [22,24,25,26]). Furthermore, since the temperature range at which *L. mulleri* seeds can germinate has widened from a medium range to include both low and high temperatures, the seeds exhibit a type 3 non-deep PD [7].

## 4. Materials and Methods

### 4.1. Study Species

*Linum mulleri* is a perennial suffrutescent plant, which flowers between May and June and bears fruit between June and July. The fruit is a capsula globose type, and the seeds are elliptical and flat [27]. *L. mulleri* is a Sardinian endemic that grows only in the Iglesiente biogeographic subsector, distributed in only three main localities: Miniera di San Giovanni di Bindua, Miniere di Monteponi, and Monte Marganai [46]. It is a xerophilous species that grows in glareicolous and garrigue environments, in poor or embryonic soils, and in the cracks of rock walls; it is found mainly on metamorphic substrates, on limestones, and in mining dumps characterized by high concentrations of heavy metals; it sometimes behaves like a pioneer species, colonizing mine tailings landfills [27]. It is a characteristic taxon of the *Polygalo sardoae*-*Linetum mulleri* community, rich in endemics and found near mines on steep rocky slopes composed of Paleozoic metalliferous limestones [47].

### 4.2. Seed Lot Collection and Preparation

Fruits containing ripe, fully developed, and healthy seeds (detected preliminarily in situ via a cut test) were collected during the time of natural dispersal in late June 2022 (Figure 1A) from the Miniera di San Giovanni di Bindua locality (39.306746° N, 8.489168° E), municipality of Iglesias. The collected seed lot was stored under controlled conditions (20 °C and 40% relative humidity) for two weeks at the Sardinian Germplasm Bank (BG-SAR) of the University of Cagliari before the germination tests were performed [48]. The seeds were cleaned manually, removing all foreign matter by hand. Additionally, the seeds were processed for five minutes in an Agriculex CB-1 Column Blower (Agriculex Inc., Guelph, Canada) to remove any empty seeds.

As the effects of and viability under chemical disinfection for seed germination of *L. mulleri* are unknown, meticulous cleaning and selection of seeds were performed to ensure high-quality material for subsequent experiments (Figure 1B).

### 4.3. Controlled Laboratory Experiments

#### 4.3.1. Germination Tests

The tests were conducted using 7000 seeds. The collection of such a large quantity of seeds was possible thanks to the remarkable capacity of the plant to produce them, as it generates thousands of seeds every year. Such abundance allows us to collect from 10 to 30% of available mature seeds without compromising the natural population, as suggested by Ref. [49], at the same time ensuring the long-term conservation of different seed lots at the Sardinian Germplasm Bank (BG-SAR) of the University of Cagliari [48]. This approach ensures that our experiments were conducted in a sustainable way, minimising any negative impact on the natural population and the conservation of the species. Tests began within two weeks after fruit collection, and to investigate the ecophysiology of the germination of *L. mulleri*, four replicates of 25 seeds were sown on the surface of 1% agar water in 60 mm diameter plastic Petri dishes (Figure 1C). A germination substrate composed of agar, dissolved slowly in hot water until it formed a viscous solution, was prepared under a laminar flow hood (FASTER, Mod. KBM, Italy) previously sterilized with UV light. Once the agar solution cooled and formed a stiff gel, the seeds were sown in the substrate using laboratory tweezers that had been sterilized at a high temperature (Figure 1C). After sowing the seeds, the Petri dishes were immediately sealed with Parafilm (Figure 1D). The seeds were incubated in growth chambers (Sanyo MLR-351/350; SANYO Electric, Osaka, Japan) using white fluorescent lamps (FL40SS.W/37 70–10 µmol m^−2^ s^−1^, Sanyo, Osaka, Japan), at constant (5, 10, 15, 20, 25, and 30 °C) and alternating temperatures (25/10 °C), under light (12 h light/12 h dark) conditions (hereafter, control, CTR). In the alternating temperature regime, the 12 h light period coincided with the elevated temperature period. The temperature conditions used under the alternating regime correspond to the mean diurnal temperature fluctuations present in Sardinia [50,51]. Furthermore, the following pre-treatments were applied: (i) cold stratification (C); (ii) warm stratification (W); (iii) warm stratification followed by cold stratification (W+C); (iv) cold stratification followed by warm stratification and another cold stratification period (C+W+C); (v) dry after-ripening (DAR) (see details in Table 5). Then, the seeds from each pre-treatment were incubated under light (12 h light/12 h dark) under the temperature regimes mentioned above.

In order to evaluate the effect of photoperiod on seed germination, four replicates of 25 seeds each were incubated in growth chambers at constant (5, 10, 15, 20, 25, 30 °C) and alternating (25/10 °C) temperatures in total darkness (0 h light/24 h dark) by wrapping dishes in two layers of aluminium foil (Table 5).

Germination, defined as visible radicle emergence (>1 mm), was recorded three times per week. Germination was monitored by marking the location of each germinated seed on the outside bottom of the Petri dish with a permanent marker (Figure 1E). The number of germinated seeds per day was simultaneously recorded on a separate worksheet. When it was necessary to remove germinated seeds and/or replace Petri dishes, the dishes were opened under laminar flow to prevent external contamination. Following germination, when the radicles reached approximately 0.2 to 0.5 mm long, the seeds were immediately transferred to a soil substrate to ensure a supply of plant material for subsequent in situ translocation (Figure 1F,G). Root length was therefore not assessed in the present study. At the end of the germination tests (for a maximum of 120 days), when no additional germination had occurred for two weeks, a cut test was carried out to determine the firmness of the remaining seeds, and the number of empty seeds was assessed via subsequent observation of the seed endosperm under a binocular microscope. Imbibed, firm seeds with white endosperm were classified as viable, while soft seeds were deemed non-viable. All conducted germination experiments were initiated at the same time in the laboratories of BG-SAR.

#### 4.3.2. Effect of Gibberellic Acid (GA_3_) on Seed Germination

According to Ref. [24], GA_3_ at concentrations of 250, 500, and 1000 mg/L has shown a positive effect on breaking seed dormancy in *L. olympicum*. Based on these results, to determine the effect of GA_3_ on seed germination in *L. mulleri*, four replicates of 25 seeds were sown in 60 mm plastic Petri dishes with 1% agar water substrate and GA_3_ at different concentrations (0, 250, 500, and 1000 mg/L GA_3_) (Table 5) and incubated under light conditions (12 h light/12 h dark), using both constant (5, 10, 15, 20, 25, and 30 °C) and alternating (25/10 °C) temperature regimes. At the end of the test, the firmness of the remaining seeds was determined, as previously detailed.

### 4.4. Data Analysis

The germination percentage (GP) was calculated as the mean of the four replicates (±SD) on the basis of the total number of filled seeds (empty seeds were excluded). Additionally, the germination rate (T_50_) was calculated as the time in days required to reach 50% germination. Generalised linear models (GLMs) were applied to (i) evaluate the effect of pre-treatments, photoperiods, and temperatures on the GP; (ii) compare the germination rates and assess the effect of pre-treatments on T_50_; (iii) evaluate the effect GA_3_ on the GP. Significant differences were then analysed using a post hoc pairwise comparison *t*-test (with Bonferroni adjustment). The GLMs, with a logit link function and quasi-binomial error structure, were used to analyse the germination percentages, while a log link function and quasi-Poisson error structure were used for analysing T_50_. Quasi-Poisson and quasi-binomial error structures and *F* tests, with an empirical rather than a Chi-squared scale parameter in the subsequent analysis of variance (ANOVA), were used to overcome the residual overdispersion [52]. All statistical analyses were performed using R v. 3.0.3 [53].

## 5. Conclusions

The results presented in this study provide useful information for both ex situ and in situ conservation of this threatened and European-protected species. Testing 7000 seeds helped to identify the dormancy type; in fact, this study allowed for the discovery that the seeds of *L. mulleri* exhibit a type 3 non-deep PD, and that this species shows the typical Mediterranean germination syndrome, considering that the highest germination percentages were recorded at temperatures of 15 °C and 20 °C. Additionally, it was detected that W, DAR, and GA_3_ (specifically, a concentration of 250 mg/L) stimulated the germination at lower (5 and 10 °C) and higher (25 and 30 °C) temperatures. Information regarding its seed germination strategies is critical for optimizing the timing and determining the success of in situ conservation efforts, such as translocation and environmental recovery. As other studies have shown (e.g., [42,54]), knowing how to germinate the seeds is vital for developing effective procedures and protocols for promoting ex situ conservation for rare and threatened species.

## Figures and Tables

**Table 1:** GLM results of germination percentage (GP) in *Linum mulleri* seeds, depending on the following factors: treatment and pre-treatment (CTR, C, W, W+C, C+W+C, DAR, dark), incubation temperature (5, 10, 15, 20, 25, 30, and 25/10 °C), and their interactions (Tr × T).

	Df	Sum Sq	Mean Sq	F	*p* Value
Treatment and Pre-Treatment (Tr)	6	4.026	0.6711	36.916	<0.001
Temperature (T)	6	11.714	1.952	107.399	<0.001
Tr × T	36	3.243	0.090	4.956	<0.001

**Table 2:** Pairwise comparisons of different treatments and pre treatments (CTR, C, W, W+C, C+W+C, DAR, dark) applied in *Linum mulleri* seeds.

	C	C+W+C	CTR	DAR	Dark	W
C+W+C	1					
CTR	0.069	<0.001 ***				
DAR	<0.05 *	<0.001 ***	1			
Dark	1	0.253	0.737	0.317		
W	0.099	<0.001 ***	1	1	0.974	
W+C	1	1	0.074	<0.05 *	1	0.106

*** *p* <0.001; * *p* < 0.05.

**Table 3:** GLM results of germination percentage (GP) in *Linum mulleri* seeds, depending on the following factors: concentration (0, 250, 500, 1000 mg/L of GA_3_), incubation temperature (5, 10, 15, 20, 25, 30, and 25/10 °C), and their interactions (Co × T).

	Df	Sum Sq	Mean Sq	F	*p* Value
Concentration (Co)	3	1.154	0.384	19.127	<0.001
Temperature (T)	6	4.498	0.749	37.275	<0.001
Co × T	18	1.037	0.057	2.866	<0.001

**Table 4:** The germination rate (T_50_) expressed in days across each tested temperature of all treatments and pre-treatments (CTR, W, C, W+C, C+W+C, DAR, 250, 500, 1000 mg/L of GA_3_) for which a 50% germination rate was achieved.

Treatment	Temperature						
	5	10	15	20	25	30	25/10
CTR	-	-	9 ± 1	8 ± 4	56 ± 44	-	37 ± 5
W	-	7 ± 2	5 ± 0	14 ± 16	100 ± 21	117 ± 3	-
C	-	-	-	55 ± 14	125 ± 71	-	-
W+C	-	-	72 ± 42	63 ± 5	-	-	-
C+W+C	-	-	31 ± 2	60 ± 8	-	-	-
DAR	-	12 ± 1	8 ± 1	8 ± 2	42 ± 12	-	30 ± 9
GA_3_ 250 mg/L	-	16 ± 1	8 ± 1	6 ± 0	15 ± 5	32 ± 6	14 ± 3
GA_3_ 500 mg/L	65 ± 24	15 ± 1	9 ± 1	8 ± 2	19 ± 5	36 ± 1	17 ± 1
GA_3_ 1000 mg/L	-	16 ± 2	11 ± 1	8 ± 1	16 ± 2	-	18 ± 11

**Table 5:** Experimental design for *Linum mulleri*. After the C, W, W+C, C+W+C, and DAR pre-treatments, the seeds were incubated at all tested temperatures (5, 10, 15, 20, 25, 30 °C, and 25/10 °C).

Treatment and Pre-Treatment	Description
CTR	Light condition (12 h light/12 h dark) and incubation at all tested temperatures
C	3 months at 5 °C
W	3 months at 25 °C
W+C	3 months at 25 °C, followed by 3 months at 5 °C
C+W+C	3 months at 5 °C, followed by 3 months at 25 °C, followed by 3 months at 5 °C
DAR	3 months at 25 °C inside a sealed glass container with colour-changing silica gel at a ratio of seed/silica gel of 1:1, which ensured the dry condition
0/24	24 h of dark conditions and incubation at all tested temperatures
GA_3_	0, 250, 500, 1000 mg/L GA_3_ in germination medium and under incubation at all tested temperatures

## Data Availability

The raw data supporting the conclusions of this article will be made available by the authors on request.
